# Immature granulocyte and neutrophil-to-lymphocyte ratio in acute appendicitis: new markers to strengthen diagnosis

**DOI:** 10.1590/1806-9282.20242063

**Published:** 2025-06-16

**Authors:** Nazlı Karakuş Kenan, Emine Doğan, Birsen Ertekin, Fatih Cemal Tekin

**Affiliations:** 1 Konya City Hospital, Department of Emergency Medicine – Konya, Türkiye.; 2 Beyhekim Training and Research Hospital, Department of Emergency Medicine – Konya, Türkiye.

**Keywords:** Acute appendicitis, C-reactive protein, Laboratory markers, Neutrophil, Lymphocyte, Granulocytes

## Abstract

**OBJECTIVE:**

The aim of this study was to assess the diagnostic accuracy of immature granulocyte count and neutrophil-to-lymphocyte ratio in acute appendicitis against traditional markers like white blood cell count and C-reactive protein.

**METHODS:**

A total of 169 appendicitis patients (appendicitis group) and 169 controls with nonspecific abdominal pain (control group) were compared in a retrospective case–control study at a single-center tertiary hospital. The analysis included demographic and laboratory data, such as immature granulocyte count, neutrophil-to-lymphocyte ratio, white blood cell, and C-reactive protein. The diagnostic accuracy of the biomarkers was assessed using receiver operating characteristic analysis.

**RESULTS:**

Compared to the control group, patients in the appendicitis group showed statistically significantly higher white blood cell, neutrophil, C-reactive protein, immature granulocyte count, immature granulocyte percentage, and neutrophil-to-lymphocyte ratio levels (p<0.001 for all). The diagnostic accuracy was highest for C-reactive protein (area under the curve: 0.994, sensitivity and specificity: 95.9%), followed by neutrophil counts (area under the curve: 0.868) and white blood cell (area under the curve: 0.862). Good diagnostic performance was observed for immature granulocyte count (area under the curve: 0.807) and neutrophil-to-lymphocyte ratio (area under the curve: 0.829).

**CONCLUSIONS:**

Immature granulocyte count and neutrophil-to-lymphocyte ratio show a significant elevation in acute appendicitis, proving useful additions to traditional diagnostic tests. C-reactive protein is the most reliable indicator; however, immature granulocyte and neutrophil-to-lymphocyte ratio may prove useful in ambiguous situations, potentially decreasing dependence on imaging techniques. Prospective studies are warranted to validate these findings and explore their prognostic value in complicated appendicitis.

## INTRODUCTION

Acute appendicitis (AA) is one of the most common causes of acute abdominal pain requiring surgical intervention. An estimated 7% of the population will experience this in their lifetime, with most cases occurring between 10 and 30 years old^1^. The difficulty of diagnosing AA, a prevalent condition, is heightened in atypical presentations, where delayed or missed diagnoses risk severe complications, such as perforation and peritonitis^2^.

Clinically diagnosing AA traditionally involves a combination of the patient's history, physical examination, and laboratory findings. Typical symptoms comprise right lower quadrant abdominal pain, fever, nausea, and vomiting^3^. However, these symptoms lack specificity and can be similar to those of other gastrointestinal and gynecological problems. Diagnostic support often includes checking laboratory values, such as white blood cell (WBC) and C-reactive protein (CRP) levels. Despite their utility, these markers lack the needed sensitivity and specificity to reliably diagnose AA, especially early in the disease process^4^.

The potential significance of novel inflammatory markers, including immature granulocyte (IG) count and neutrophil-to-lymphocyte ratio (NLR), is highlighted by recent advances in laboratory diagnostics^5–7^. IGs, granulocytes newly released by the bone marrow, indicate bone marrow activity during systemic inflammation^5^. Likewise, NLR, the ratio of absolute neutrophil count and lymphocyte count, has proven to be a straightforward and affordable indicator of systemic inflammation. These two parameters have demonstrated efficacy in distinguishing inflammatory from noninflammatory conditions across various clinical settings^5,7,8^.

Studies on AA have explored the diagnostic value of IG and NLR, showing varying levels of success. Elevated IG levels were significantly associated with AA compared to nonspecific abdominal pain in studies, hinting at its potential as a diagnostic marker^9,10^. Hajibandeh et al.'s meta-analysis also showed that NLR could be a valuable supplementary diagnostic test for AA, especially when the diagnosis is unclear^7^. The clinical applicability of these markers is questionable despite these findings, with some studies showing limited diagnostic accuracy.

Imaging modalities such as ultrasonography and computed tomography (CT) are often employed to confirm the diagnosis of AA. While highly effective, these techniques have limitations, including cost, availability, and potential risks associated with radiation exposure^11^. Therefore, to improve patient outcomes and reduce the need for imaging, there is a growing focus on finding reliable and noninvasive biomarkers for early and accurate AA diagnosis.

This study seeks to determine the diagnostic value of IG count and NLR in AA by comparing them against traditional markers like WBC count and CRP. This study investigates the potential clinical value of these parameters when diagnosing AA and nonspecific abdominal pain in a defined patient group. Furthermore, this research examines the possibility of using these markers within diagnostic algorithms to improve both the accuracy and efficiency of AA diagnosis.

## METHODS

### Study design and participants

A retrospective, single-center, case–control study was performed at the Emergency Medicine Clinic of Konya City Hospital. The Meram Medical Faculty review board of Necmettin Erbakan University granted ethical approval (approval no. 2024/4749). All patients with intact cognition, and the proxies of those with impaired cognition, provided informed consent. This research was conducted in accordance with the Helsinki World Medical Association Declaration.

The appendicitis group (AG) included adult patients (≥18 years) with AA who were admitted to the emergency department of Konya City Hospital from August 2020 to April 2022. Adults aged over 18 years with unspecified abdominal pain, presenting to the emergency department, formed the control group (CG). A total of 338 patients participated in the study, with 169 patients assigned to each of the two groups.

The study excluded patients under 18 years of age, pregnant participants, those with chronic inflammatory/oncological/hematological/immunological diseases, immunocompromised patients, patients who applied with nonspecific abdominal pain and received a diagnosis other than AA, patients with inaccessible records, and those who left against medical advice.

We retrospectively reviewed patient records—epicrisis reports and the electronic health record system—to collect admission data at the time of admission of patients diagnosed with AA in the emergency department between the specified dates. This included age, sex, and laboratory results, including WBC, neutrophils, lymphocytes, NLR, CRP, IG counts, and IG percentages. Automated analyzers, following standardized procedures, performed the laboratory measurements.

The primary outcome of this study is to determine the effect of IG levels on the diagnosis of AA. The secondary outcomes are to investigate how IG and NLR are linked to other inflammatory markers, the diagnostic potential of NLR for AA, and the differences in IG and NLR ratios between patients discharged with nonspecific abdominal pain and those hospitalized with AA.

### Statistical analysis

The statistical analyses were performed with IBM SPSS 23.0 (IBM SPSS Statistics, Version 23.0, IBM Corp. Armonk, NY, USA). Medians and interquartile ranges (IQRs) showed continuous variable data, and frequencies and percentages showed categorical variable data. The Mann-Whitney U and chi-square tests analyzed continuous and categorical variables, respectively. The diagnostic accuracy of laboratory parameters was evaluated using receiver operating characteristic (ROC) curve analysis. Calculations were performed to determine the area under the curve (AUC), sensitivity, specificity, and optimal cutoff values. The optimal cutoff point was established using the Youden index. The results were considered statistically significant if p<0.05.

## RESULTS

### Comparison of parameters between appendicitis group and control group

Table 1 presents a summary of the demographic and laboratory features of the AG and CG. No age distribution differences were found between the groups (p=0.257). The AG had a statistically significantly higher male proportion (70.4 vs. 43.8%, p<0.001).

**Table 1:** Comparison of parameters between appendicitis and control groups

Variables		Appendicitis group, n (%), n=169	Control group, n (%), n=169	p-value
Age (years)	18–40	113 (66.9)	103 (60.9)	0.257
	40–65	56 (33.1)	66 (39.1)	
Sex	Male	119 (70.4)	74 (43.8)	**<0.001**
	Female	50 (29.6)	95 (56.2)	
WBC count (×10^3^ **μ** L)		13.4 (11.1–16.6)	8.5 (6.8–10.4)	**<0.001**
Lymphocyte count (×10^3^ **μ**L)		1.8 (1.2–2.5)	2.2 (1.7–2.8)	**<0.001**
Neutrophil count (×10^3^ **μ**L)		10.5 (7.7–14)	5.0 (3.7–7)	**<0.001**
NLR		5.8 (3.7–11.7)	2.7 (1.6–3.2)	**<0.001**
CRP (mg/L)		48.6 (19.6–101.1)	1.6 (0.7–3.1)	**<0.001**
IG count (×10^3^ **μ**L)		0.06 (0.04–0.08)	0.02 (0.02–0.04)	**<0.001**
IG%		0.4 (0.3–0.5)	0.3 (0.2–0.4)	**<0.001**

Numeric variables are presented as median (IQR: Q1–Q3), and categorical variables are presented as n (percentage). CRP: C-reactive protein; IG: immature granulocyte; IG%: Immature granulocyte percentage; IQR: interquartile range; NLR: neutrophil-to-lymphocyte ratio; WBC: white blood cell. Statistically significant values are denoted in bold.

The AG showed significantly higher median WBC (13.4×10³/μL vs. 8.5×10³/μL), neutrophil (10.5×10³/μL vs. 5.0×10³/μL), and NLR (5.8 vs. 2.7) compared to the CG (for all p<0.001). In contrast, the median lymphocyte count was statistically significantly reduced in the AG (1.8×10³/μL vs. 2.2×10³/μL, p<0.001). Statistically significantly increased median CRP (48.6 mg/L vs. 1.6 mg/L), IG count (0.06×10³/μL vs. 0.02×10³/μL), and IG percentage (0.4 vs. 0.3%) were observed in the AG (for all p<0.001).

### Receiver operating characteristic analysis for the prediction of acute appendicitis

The ability of several laboratory parameters to diagnose AA was evaluated via ROC analysis (Table 2). CRP showed excellent diagnostic accuracy, achieving an AUC of 0.994 (95%CI 0.994–0.999). Using >8.7 mg/L as a CRP cutoff yielded 95.9% sensitivity and specificity.

**Table 2:** The receiver operating characteristic analysis of parameters in the prediction of acute appendicitis

Risk factor	AUC	%CI	p-value	Cutoff	Sensitivity (%)	Specificity (%)
CRP (mg/dL)	0.994	0.994–0.999	<0.001	>8.7	95.9	95.9
Neutrophil count (×10^3^μL)	0.868	0.830–0.906	<0.001	>7.3	79.9	78.1
WBC count (×10^3^μL)	0.862	0.822–0.901	<0.001	>10.6	79.9	78.7
NLR	0.829	0.785–0.872	<0.001	>3.3	78.7	77.5
IG count (×10^3^μL)	0.807	0.761–0.853	<0.001	>0.035	75.7	73.4
IG%	0.682	0.625–0.739	<0.001	>0.35	61.5	64.5
Lymphocyte count (×10^3^μL)	0.634	0.574–0.693	<0.001	<2.04	62.7	59.2

AUC: area under the curve; CI: confidence interval; CRP: C-reactive protein; IG: immature granulocyte; IG%: immature granulocyte percentage; NLR: neutrophil-to-lymphocyte ratio; WBC: white blood cell.

**Figure 1:**
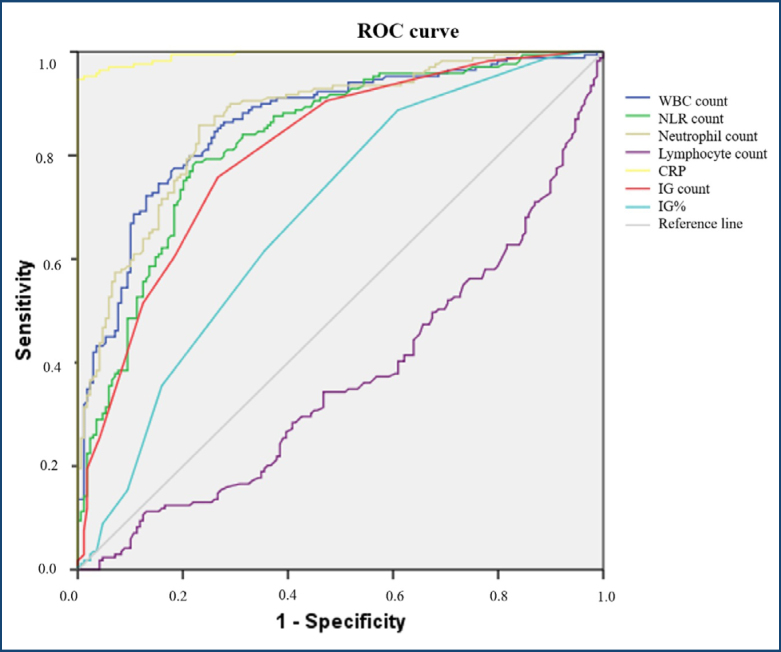
Receiver operating characteristic curve analysis of laboratory parameters. ROC: receiver operating characteristic; WBC: white blood cell; NLR: neutrophil-to-lymphocyte ratio; CRP: C-reactive protein; IG: immature granulocyte; IG%: immature granulocyte percentage.

The diagnostic performance of neutrophil and WBC counts was good, yielding AUCs of 0.868 (95%CI 0.830–0.906) and 0.862 (95%CI 0.822–0.901, p<0.001), respectively. The optimal cutoff values for neutrophil count and WBC counts were >7.3 × 10³/μL and >10.6 × 10³/μL, yielding sensitivities of 78.7% and 79.9% and specificities of 78.1% and 78.7%78.1 and 78.7%, respectively (Figure 1).

Additional parameters like NLR and IG count showed good diagnostic accuracy, with AUCs of 0.829 and 0.807, respectively (p<0.001 for all). The IG percentage poorly predicted AA (AUC: 0.682, p<0.001). The lymphocyte count showed the poorest diagnostic reliability, recording an AUC of 0.634 (95%CI 0.574–0.693).

## DISCUSSION

The results of the current study highlighted that IG count, IG percentage, and NLR are significantly elevated in patients with AA compared to those with nonspecific abdominal pain, although their diagnostic accuracy is good compared to established markers, such as CRP.

The findings reveal that IG count and IG percentage are significantly higher in the AG than in the CG. This supports previous research showing that increased IG levels indicate a more active bone marrow responding to acute inflammation and infection^6,9^. IG levels, proposed as reliable markers, correlate with the severity of systemic inflammation and sepsis. For instance, research by Ansari-Lari et al.^5^ showed that higher IG levels correlated with poorer results for those with sepsis. The current findings show that these observations also hold true for AA.

Consistent with previous studies^7,12^, significantly higher NLR in the AG reinforces its role as a practical marker to distinguish AA from nonspecific abdominal pain. The NLR is a key indicator of systemic inflammation, reflecting the opposing forces of neutrophil-driven and lymphocyte-driven immune processes^8^. Our findings, showing an optimal NLR cutoff with good diagnostic accuracy, support Hajibandeh et al.'s meta-analysis^7^, which highlights NLR's diagnostic utility in AA.

The promising diagnostic performance of IG and NLR indicates their potential value as additional markers, not as stand-alone diagnostic tests. Unal's study reported fair AUC performance for IG count, IG percentage, and NLR in differentiating between AA and non-AA individuals^13^. Predicting complicated appendicitis proved even more accurate using the AUC values of IG count and IG percentage in that study^13^. However, different studies found that the AUC values for IG count and IG percentage were fair^14,15^. Park et al.^15^ reported the lowest AUC value for IG percentage in predicting AA. Compared to other inflammatory markers, the AUC value for IG percentage was lower in this study. This research suggests that IG percentage is an inferior predictor of inflammation when compared to alternative methods. Population variations, differing study conditions (abdominal pain-AA, complicated AA, perforated AA, etc.), and inconsistent ROC cutoff calculations are the key reasons for study discrepancies. When traditional markers and clinical observations are inconclusive, these parameters prove particularly valuable. Furthermore, integrating them into diagnostic algorithms could lessen our dependence on expensive and potentially harmful imaging techniques, such as ultrasounds and CT scans^11^.

While IG and NLR were statistically significant, CRP showed better diagnostic performance, having the highest AUC value on ROC analysis. The acute-phase reactant CRP, synthesized in response to interleukin-6, serves as the gold-standard inflammatory marker for diagnosing AA^16^. Güngör et al. reported that the highest AUC value for predicting complex AA was linked to CRP levels^17^. In the present study, using >8.7 mg/L as a CRP cutoff yielded 95.9% sensitivity and specificity, while this cutoff value is lower than the other studies^16,17^. The present study results confirm prior research^18^ showing high diagnostic accuracy, sensitivity, and specificity in identifying acute intra-abdominal pathologies. While diagnostic accuracy is high for WBC and neutrophil counts, their lack of specificity (compared to CRP) is because of elevated counts under various inflammatory conditions, limiting their use for AA identification^18^. Incorporating IG and NLR alongside traditional markers might improve diagnostic accuracy through an additional understanding of inflammation. Moreover, it is crucial to understand these markers within their clinical context; comorbidities, medications, and individual physiological differences affect their levels. Specifically, immunosuppressive therapy or chronic inflammatory diseases might reduce neutrophil activity, resulting in inaccurate negative test results^19^.

Several limitations of this study must be acknowledged. First, using a retrospective design inherently introduces biases, such as incomplete data and selection bias. Second, the findings of this single-center study may not be generalizable to other populations and healthcare settings. Third, to confirm their clinical utility, IG and NLR markers require validation from larger, prospective, multicenter studies.

The roles of IG and NLR in predicting disease severity and outcomes in AA warrant further investigation. For instance, studies have shown a correlation between increased IG levels and perforated appendicitis, suggesting their value in prognosis^13,14,20^. In addition, combining IG and NLR data with newer biomarkers, for instance, procalcitonin or interleukin-6, might further boost diagnostic precision.

## CONCLUSION

Increased IG count and NLR are strongly associated with AA and offer a good level of diagnostic accuracy. Despite not exceeding established markers like CRP, these markers provide useful additional information to assist in early AA diagnosis and treatment. Therefore, including them in clinical practice may lead to more accurate diagnoses and better patient results. However, further research is necessary to confirm their practical value, define standardized cutoff points, and assess their prognostic significance.
